# Temperature Effects on the Survival and Oviposition of an Invasive Blow Fly *Chrysomya rufifacies* Macquart (Diptera: Calliphoridae)

**DOI:** 10.3390/insects16030310

**Published:** 2025-03-17

**Authors:** Travis W. Rusch, Samantha J. Sawyer, Abigail E. Orr, Nicholas Richter, David Sohn, Lauren Gagner, Alexandria Smith, Jeffery K. Tomberlin, Aaron M. Tarone

**Affiliations:** Department of Entomology, Texas A&M University, College Station, TX 77843, USA; travis.rusch@usda.gov (T.W.R.); ssawyer@newhaven.edu (S.J.S.); abelor@tamu.edu (A.E.O.); nwr12@tamu.edu (N.R.); dcsohn@gmail.com (D.S.); lng021@tamu.edu (L.G.); abs8991@tamu.edu (A.S.); jeffery.tomberlin@ag.tamu.edu (J.K.T.)

**Keywords:** acute stress, bet-hedging, reproduction, thermal performance, thermal tolerance

## Abstract

The globally increased severity and frequency of elevated temperatures are altering native species’ geographic distributions and local abundances while also increasing the invasion of new areas by exotic species. These distributional shifts have affected native species. Through two experiments, we investigated the effects of temperature on the survival and oviposition of the hairy maggot blow fly *Chrysomya rufifacies* (Macquart), a highly competitive and predatory invasive blow fly of ecological, economic, and forensic importance. In our first experiment, we exposed mixed-sex colonies of *C. rufifacies* to a given temperature (10–45.0 °C) for 24 h. High survival (≥90%) was observed from 10 to 40 °C, with moderate mortality at 42.5 °C (29.2%) and high mortality at 43.5 °C (75.4%). All flies died when exposed to 44.5 or 45.0 °C for 24 h. Oviposition occurred from 22.5 to 42.5 °C, with the greatest occurrences (100%) at 30 and 35 °C and the greatest number of eggs (2035) occurring at 30 °C. Although oviposition occurred from 22.5 to 42.5 °C, egg viability was only observed from 22.5 to 37.5 °C. Thus, *C. rufifacies* has distinct thermal limits for survival, and oviposition may exhibit a bet-hedging strategy in response to temperature exposure. In our second experiment, we assessed the effects of an acute heat shock on *C. rufifacies* oviposition performance. Adult virgins (males and females) were exposed to 25.0 °C, 42.0 °C, or 44.0 °C for 1 h, and then maintained at ~25 °C in mixed-sex colonies for 14 d. Pre-breeding heat exposure had no effect on male or female reproductive success, except for females exposed to 44.0 °C. Females exposed to this temperature before breeding oviposited sooner (2.5 ± 0.0 d, 37.5% decrease), more frequently (0.5 ± 0.4, 33.3% increase), and produced more eggs (10,772.9 ± 2258.6 eggs, 73.3% increase) than female flies exposed to 25 °C. The combined results show that *C. rufifacies* survives exposures up to 43.5 °C, successfully oviposits up to 37.5 °C, and accelerates both oviposition timing and intensity following brief exposure to near upper lethal temperatures (44.0 °C), potentially provides *C. rufifacies* a competitive advantage over native calliphorids in warming environments.

## Simple Summary

The hairy maggot blow fly (*Chrysomya rufifacies*) is an invasive species in North America that can outcompete and displace multiple native blow fly species. However, their survival and reproduction are largely limited by environmental temperature. Through two laboratory experiments, we investigated the effects of temperature on the reproduction and survival of *Chrysomya rufifacies*. First, we quantified the effects of temperature on egg laying by exposing groups of flies to a range of temperatures (10–45 °C) for 24 h and quantifying both the probability of egg laying and the masses of eggs produced. We also quantified the probability of fly survival when exposed to a constant temperature (24 h at 10–45 °C). Next, we quantified the effects of acute heat shock (1 h exposure) on flies and found the strongest heat shock treatment (44 °C) caused females to lay eggs sooner, more frequently, and more in total than females exposed to control (25 °C) or mild (42 °C) acute heat shock treatments. These data are important in determining when and where this invasive blow fly can be active and reproducing, which will help determine its potential impact on native blow fly species. Additionally, forensic entomologists can use these data in death investigations to help inform time of colonization estimates, which can ultimately help determine the postmortem time interval (i.e., time of death) given certain assumptions.

## 1. Introduction

Organisms regularly experience daily and seasonal temperature fluctuations that result in periodic exposures to thermally stressful conditions [1,2,3]. However, the likelihood that any organism will endure periods of thermal stress is increasing as global heatwaves are becoming “the new normal” due to climate change [4,5,6]. Small-bodied ectotherms are particularly vulnerable to heatwaves as their body temperatures largely rely on environmental temperatures [7,8,9]. For individuals, heat waves limit activity times [2,9,10], reduce organismal performances [11,12,13], and lower reproductive potential [14,15,16]. For populations, heatwaves reduce species abundances, disrupt population dynamics, and restrict species distributions [17,18,19]. Such findings have stimulated concern and debate on a potential “insect apocalypse” in response to the rising temperatures of climate change [20,21,22].

While not all temperatures experienced during heatwaves are lethal, brief (i.e., hours to days) exposure to elevated temperatures can have lasting effects on reproduction [2,23]. For instance, elevated but sublethal temperature exposures can temporarily [24,25] or permanently [15,26,27] sterilize male insects due to sperm death or testicular damage. Similarly, egg-laying female insects may reduce or cease oviposition due to physiological limits, innate refusal, or by exhibiting thermally-dependent decisions (e.g., bet-hedging) during heatwaves [28,29,30]. To accurately predict behavioral and evolutionary reproductive responses to the elevated temperatures projected with climate change, it is critical to identify both thermal limits and thermal performances.

Through two experiments, we investigated the effects of temperature on the survival and oviposition of the hairy maggot blow fly *Chrysomya rufifacies* (Macquart) (Diptera: Calliphoridae). This species was introduced to North America in the 1970s from the Australasian regions of the Old-World Tropics [31,32]. *Chrysomya rufifacies* has ecological, economic, and forensic importance as they feed on carrion [31,32,33], thus playing an important role in nutrient recycling [34]. However, *C. rufifacies* can outcompete and displace native blow flies on carrion [32,35], posing a potentially invasive threat [36]. *Chrysomya rufifacies* also engage in livestock myiasis, resulting in economic losses [31,32], but also feed on human remains, making it a useful tool for forensic entomologists in death investigations [33,34]. As with previous blow fly studies [29,37,38], our first experiment reveals temperature-dependent oviposition performances when exposed to constant and prolonged temperatures (e.g., number of eggs laid, frequency of oviposition events, and time to first oviposition). However, because adult blow flies are highly mobile, they likely seek out thermal refuge (e.g., shade) if available during heatwaves. In doing so, they should experience elevated body temperatures for short durations (e.g., minutes–hours) when moving between microclimates, which may still affect the fecundity [39,40]. To investigate this, our second experiment exposed both adult male and female virgin *C. rufifacies* to one of three acute (1 h) temperature exposure treatments (25 °C, 42 °C, or 44 °C) before mating and recorded; (1) time to oviposition, (2) oviposition frequency, and (3) total egg mass. We predicted that oviposition performance would decline as temperature increased because sperm damage and fly death increased with temperature [15,26], consequently hindering oviposition under certain thermal conditions [29].

## 2. Methods

### 2.1. Colony Care

To initiate and maintain a laboratory colony, we collected adult *C. rufifacies* (>500) from decomposing animal remains in College Station, TX, USA from May to July 2017. Flies were identified prior to experiments using morphological features [41] (voucher #736) and held in a semi-climate-controlled room (~25 °C, 50% RH, and a 14L:10D photoperiod) in 30 cm × 30 cm × 30 cm cages (BioQuip, Rancho Dominguez, CA, USA) at Texas A&M University, USA. While in the laboratory flies were provided water and food (50:50 table sugar and milk powder) ad libitum. Flies were also provided protein meals (~5 mL beef blood) every other day for 6 d to encourage reproduction and then provided beef liver (~20 g) for an oviposition site. Eggs (*n*~250) were transferred to a glass mason jar (79 mm × 178 mm; 946 mL, Ball Inc., Daleville, IN, USA) half filled with vermiculite (Sungro Agriculture, Agawam, MA, USA), capped with a breathable cloth lid (WypAll, Kimberly-Clark Inc., Roswell, GA, USA), and held in the same room as adults. Larvae were provided additional beef liver (as needed) until pupation. Upon emergence, ~200 flies were released from jars into empty cages (colony = 8 cages, ~1600 adults). To reduce laboratory acclimation and maintain genetic diversity, we periodically added wild-caught adults to the laboratory colony. When wild-type adults were added we reset the laboratory colony back to generation zero. All flies used in the experiments were between G_4_ and G_7_.

### 2.2. Survival and Oviposition Performance

We quantified the effects of constant temperature exposure on the survival and oviposition performance of adult *C. rufifacies* (*n* = 3600; 1800 males and 1800 females). Mixed sex colonies (*n* = 3) of *C. rufifacies* (*n* = 20 males and 20 females; *n* = 40 flies per colony cage) were separated into colony cages (described above) and then placed into incubators (*n* = 6 incubators; Percival Scientific, Perry, IA, USA) preset to a temperature ranging 10–45 °C for 24 h. During experiments, each cage contained a(*n*) water, food, and oviposition source (described above). The first replicate of temperature treatments was spaced at 5 °C intervals (i.e., 10, 15, 20, 25, 30, 35, 40, and 45 °C) making eight treatment temperatures. To more accurately determine the lower and upper limits of oviposition, additional temperature treatments at narrower intervals (i.e., 12.5, 17.5, 22.5, 37.5, 42.5, 43.5, and 44.5 °C) were included, for a total of 15 temperature treatments. Each temperature treatment was replicated twice with each replicate consisting of 3 colony cages (*n* = 6 colony cages per temperature treatment; *n* = 240 flies per temperature treatment; 120 males and 120 females). After the 24 h temperature treatment, survival (i.e., number dead and alive by sex) and oviposition were quantified. If oviposition occurred, eggs were extracted from the oviposition site using a paintbrush, lightly wiped with a paper towel to remove any fluids from oviposition site, and weighed. Additionally, a subset of 20–50 eggs were reared out to test viability (i.e., hatch to first instar) at each temperature using the same methods for colony care described above. Note, these subsets of eggs were taken after egg masses were taken, and some temperature treatments resulted in only 20 eggs laid. Furthermore, the number of hatched eggs was not quantified, just if any eggs hatched, to the first instar stage within 5 days. After hatch (or no hatch after 5 days) eggs were discarded as the goal was to find the lower and upper thermal limits for egg hatch.

### 2.3. Heat Shock Exposure

We assessed the effect of acute heat stress on blow fly oviposition performance for adult *C. rufifacies* (*n* = 900) using three metrics following their acute heat exposure: (1) time to oviposition, (2) frequency of oviposition, and (3) total egg mass. Time to oviposition was defined as the time point at which eggs were first laid, measured at 12 h intervals. Frequency of oviposition was defined as the number of times eggs were present divided by 28 egg checks (measured at 12 h intervals for 14 d) and are expressed as proportions. Total egg mass was defined as the sum of all eggs found for a given treatment over the 14 d post-exposure period.

To ensure adults did not mate before experiments, pupae were separated into individual 120 mL condiment cups (SOLO Cup Company, Lake Forest, IL, USA). Upon emergence, flies were sexed and placed in single-sex cages with sugar and water provided ad libitum. After a 48 h emergence period, individual flies were transferred into breathable 1.5 mL microcentrifuge tube (Thermo Fisher Scientific, Waltham, MA, USA), and then placed in a pre-warmed incubator (Percival Scientific, Perry, IA, USA) set to either 25 °C, 42 °C, or 44 °C for 1 h.

After heat treatments, flies were transferred to a new Bugdorm containing untreated virgin flies of the same age but opposite sex (*n* = 25 males + 25 females per cage). We tested six experimental combinations: (1) 25 °C exposed males with untreated females, (2) 25 °C exposed females with untreated males, (3) 42 °C exposed males with untreated females, (4) 42 °C exposed females with untreated males, (5) 44 °C exposed males with untreated females, and (6) 44 °C exposed females with untreated males. Flies were held in the same room (described above) and supplied sugar and water ad libitum. Additionally, ~25 g of beef liver was placed in a Petri dish in each cage as both a protein source and oviposition site. All beef livers were wrapped in plastic wrap to prevent flies from getting stuck to the liver and dying during experiments. However, small holes were punctured in the plastic wrap to give flies access to the liver. During experiments, livers were checked for eggs and replaced at both 800 and 2000 h to provide a constant and consistent protein source and oviposition site. When found, eggs were removed from the liver, wiped dry, and their mass was recorded.

### 2.4. Statistical Analyses

For our survival and oviposition performance analyses, we developed global models for our a priori hypotheses using generalized additive models (GAM) with a spline fit (for smoothing purposes), using the “mgcv” package (version 1.9-1) [42,43]. For survival, we included temperature and sex, as well as their interaction as main effects [44]. For oviposition performance, we first developed a GAM model to predict the probability of oviposition across temperature exposure using temperature as a main effect. We then developed a second GAM model for oviposition performance to predict total egg mass across temperature exposure using temperature as a main effect.

For our heat shock exposure analysis, we developed global models based on our a priori hypotheses using generalized linear mixed-effects models (GLMM) using the “lme4” package (version 1.1-36) [45]. Three separate global models tested the effects of temperature, sex, and trial, as well as the interactive effects of temperature and sex, on; (1) time to oviposition, (2) frequency of oviposition, and (3) total egg mass. For all models, temperature and sex, as well as their interaction term, were set as fixed effects, while trial was set as a random effect [44]. All analyses were completed using the R Statistical Software (version 3.5.2) [46].

## 3. Results

**Figure 1:**
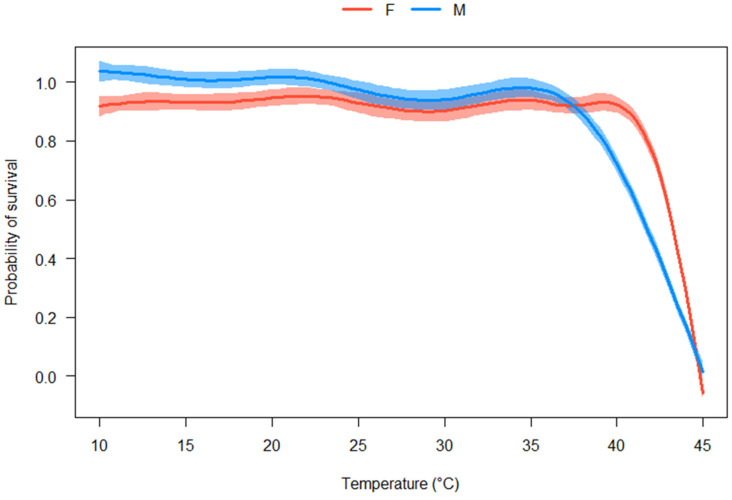
Model visualizations of a generalized additive model predicting the probability of survival at each treatment temperature ranging 10 to 45 °C. Survival was observed from 10 to 43.5 °C only (see Figure S1), with the greatest predicted probabilities of survival at 40.0 °C and below. Lines represent the model-predicted probabilities of survival for males (blue) and females (red). Margins around each line indicate 95% confidence intervals.

**Figure 2:**
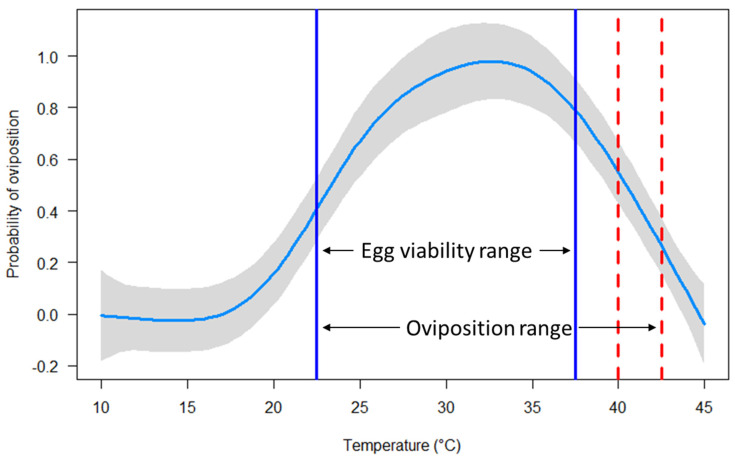
Model visualizations of a generalized additive model predicting the probability of oviposition at each treatment temperature ranging 10 to 45 °C. Oviposition occurred from 22.5 to 42.5 °C, with the greatest observed egg-laying frequencies (100%) at 30.0 and 35.0 °C (see Figure S2a,b) and the greatest model-predicted probability at 32.5 °C. However, egg viability (i.e., egg viability range, some amount of eggs hatch to first instar) was only observed from 22.5 to 37.5 °C. Light blue curved line indicates the model predicted probability of oviposition across temperatures. Grey margins indicate 95% confidence intervals. Vertical solid blue lines indicate the lowest (left) and highest (right) temperatures where eggs were viable (i.e., egg viability range). Vertical red dashed lines indicate temperatures where eggs were laid but were not viable (i.e., 40.0 and 42.5 °C). Note, no eggs were laid below viable temperatures. Therefore, the oviposition range (i.e., the total range of temperatures where any eggs, viable and non-viable, were observed to be laid) is from the furthest left vertical solid blue line (22.5 °C) to the furthest right vertical dashed red line (42.5 °C).

**Figure 3:**
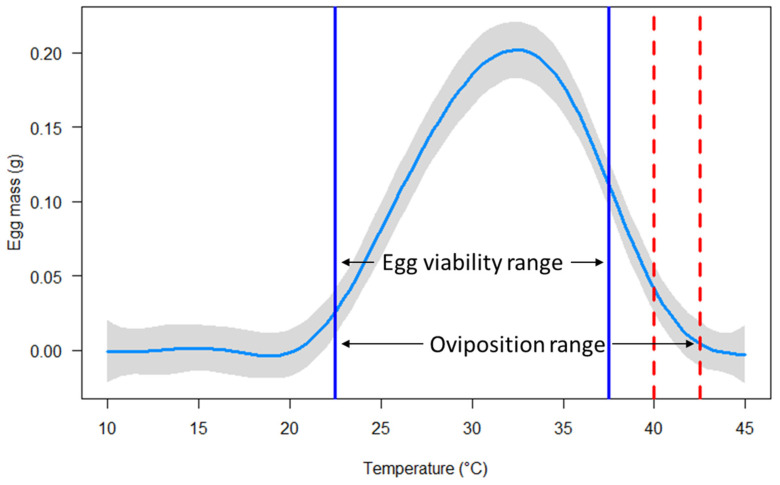
Model visualizations of a generalized additive model predicting the mean total egg masses at each treatment temperature ranging 10–45 °C. The greatest observed mean total egg masses were at 30.0 and 35.0 °C (Figure S3a,b), while the greatest model predicted egg mass was at 32.5 °C, and dropped to zero at both 20.0 and 43.5 °C. Light blue curved line indicates the model predicted probability of oviposition across temperatures. Grey margins indicate 95% confidence intervals. Vertical solid blue lines indicate the lowest (left) and highest (right) temperatures where eggs were viable (i.e., egg viability range, where some amount of eggs hatch to first instar). Vertical red dashed lines indicate temperatures where eggs were laid but were not viable (i.e., 40.0 and 42.5 °C). Note, no eggs were laid below viable temperatures. Therefore, the oviposition range (i.e., the total range of temperatures where any eggs, viable and non-viable, were observed to be laid) is from the furthest left vertical solid blue line (22.5 °C) to the furthest right vertical dashed red line (42.5 °C).

The interactive effects of temperature and sex strongly influenced the survival of *C. rufifacies* (*p* < 2 × 10^−16^). High survival (≥90%) was observed from 10 to 40 °C, with moderate mortality at 42.5 °C (29.2%) and high mortality at 43.5 °C (75.4%). All flies died when exposed to 44.5 or 45.0 °C for 24 h (Figure 1 and Figure S1). Similarly, temperature strongly determined both the probability of oviposition (*p* < 2 × 10^−16^) and the total egg mass produced (*p* < 2 × 10^−16^) by female *C. rufifacies*. Oviposition occurred from 22.5 to 42.5 °C, with the greatest probabilities (100%) at 30 and 35 °C (Figure 2 and Figure S2a,b) and the greatest average egg mass (0.22 ± 0.05 g) occurring at 30 °C (Figure 3 and Figure S3a,b). Although oviposition occurred from 22.5 to 42.5 °C (i.e., the total range of temperatures where any eggs, viable and non-viable, were observed to be laid), egg viability (i.e., some amount of egg hatch to first instar) was only observed from 22.5 to 37.5 °C (Figure S4).

**Figure 4:**
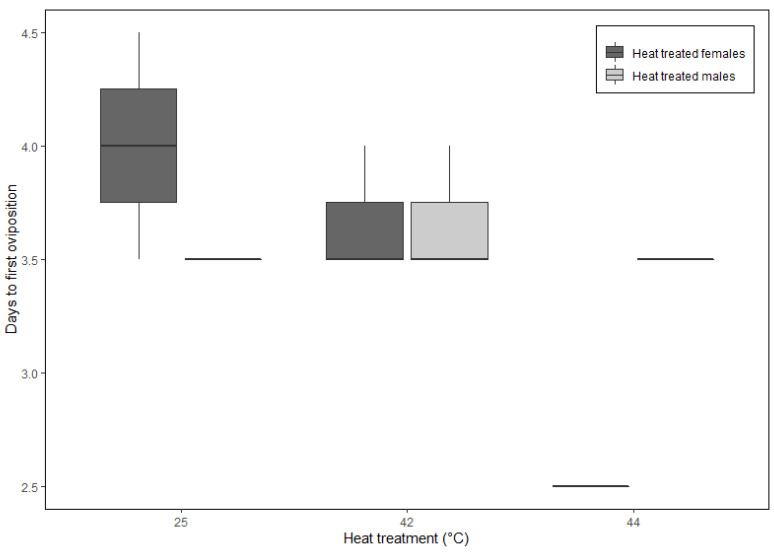
The time (days) until first oviposition was the shortest for females exposed to a pre-breeding heat shock treatment (i.e., heat-treated females) of 44 °C, compared to all other temperature treatments. Box and whisker plots display the minimum, the maximum, the sample median, and the first and third quartiles.

**Figure 5:**
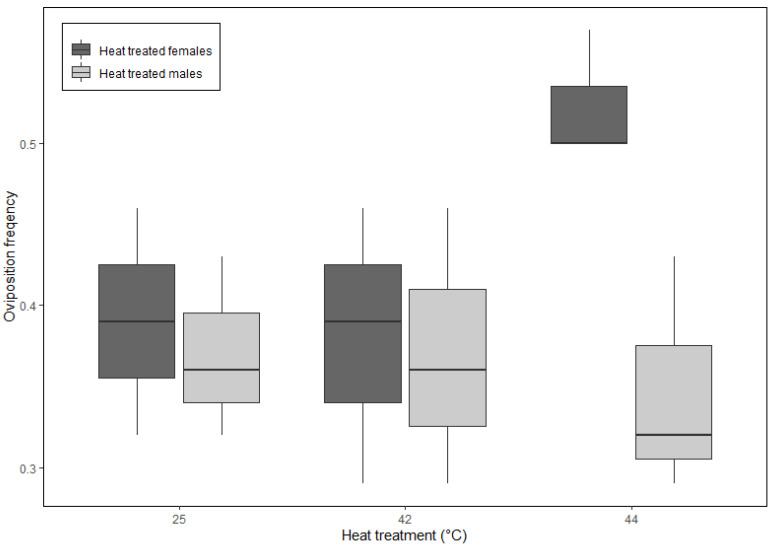
The frequency of oviposition events was greatest for females exposed to a pre-breeding heat shock treatment (i.e., heat-treated females) of 44 °C, compared to all other temperature treatments and are expressed as proportions; number of times eggs were present divided by 28 egg checks over the 14 d experimental period. Box and whisker plots display the minimum, the maximum, the sample median, and the first and third quartiles.

**Figure 6:**
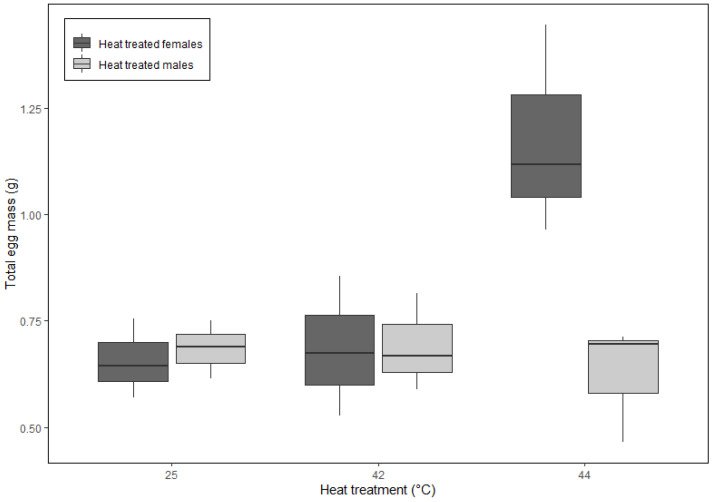
The mean total egg mass following heat treatments over a 14 d breeding period was greatest for females exposed to a pre-breeding heat shock treatment (i.e., heat-treated females) of 44 °C, compared to all other temperature treatments. Box and whisker plots display the minimum, the maximum, the sample median, and the first and third quartiles.

The interactive effects of temperature and sex strongly influenced all three oviposition behaviors (i.e., time to oviposition, frequency of oviposition, and total egg mass), while the trial had negligible effects (File S1). However, only females exposed to 44 °C exhibited notable responses; oviposited sooner (2.5 ± 0.0 d; Figure 4), more frequently (0.5 ± 0.4; Figure 5), and produced the greatest egg mass (1.18 ± 0.25 g; Figure 6).

## 4. Discussion

As in other ectotherms, environmental temperature influenced the biological processes involved in the survival and oviposition of *C. ruficacies*. All flies survived at high levels when exposed to low to moderate temperatures, but declined when temperatures surpassed 40 °C, which aligns with other blow fly studies [38,47,48]. Female *C. rufifacies* did show a greater thermal tolerance for survival than males (Figure 1 and Figure S1). This may be due to females generally being larger than males, as a larger body size can retain more nutrients (e.g., water and lipid reserves) that can be used to combat the negative effects of elevated temperatures (e.g., desiccation) [49,50,51]. Female *C. rufifacies* also displayed clear upper and lower thermal tolerances of oviposition, as well as varying levels of oviposition thermal performance (i.e., egg production) across temperatures (Figure 2, Figure 3, Figures S2 and S3). Flies were either physiologically unable to, or innately (or consciously) decided not to lay eggs below 22.5 °C or above 42.5 °C. If not a physiological limitation, flies may exhibit a bet-hedging strategy of waiting out suboptimal temperatures that could negatively affect the development and survival of their offspring for more optimal temperatures to increase their fitness in unpredictable environments [30,52,53]. Further research, especially that of fluctuating vs. constant temperature, is required to disentangle physiological limits from behavioral responses.

Our second set of experiments revealed that acute pre-mating heat stress can alter oviposition in *C. rufifacies*. While our results in the heat shock study align with a field study where a land snail (*Cepaea nemoralis*) produced more eggs more frequently following warm and dry weather events [40], there does not appear to be a universal response to acute pre-mating heat exposure. For instance, two white fly species showed contrasting results; *Trialeurodes vaporariorum* decreased the number of eggs oviposited with an increasing pre-mating heat exposure (26–45 °C for 1 h), while *Bemisia tabaci* showed no effect of pre-mating heat exposure (26–45 °C for 1 h) on total egg production [39]. Furthermore, neither whitefly species altered their oviposition timing following a 1 h heat exposure up to 45 °C [39]. Collectively, these results reveal the complex and likely species-specific, oviposition responses to pre-mating heat exposures.

To maximize fitness, egg-laying insects could engage in a bet-hedging strategy [30] where they modify their oviposition timing and site selection in relation to the environmental quality best suited for their offspring [54,55,56]. If *C. rufifacies* females interpreted their pre-mating thermal environment as stressful (i.e., low quality), they may have accelerated their oviposition timing to take advantage of less stressful temperatures (i.e., the constant 25 °C post-treatment temperatures) as they cannot predict if or when suboptimal conditions would occur again. Our acute heat treatment experiment brings up the question of how *C. rufifacies* (and other organisms) might respond to continued fluctuating temperatures. While there is no published data that we are aware of for the effects of fluctuating temperatures on the oviposition paper of *C. rufifacies*, another study [57] found opposite results to ours, that codling moths (*Cydia pomonella* (L.) (Lepidoptera: Tortricidae)) decreased its egg output when exposed to fluctuating (compared to constant) temperatures. Thus, if our treatment persisted, and consequently became a repeated and thus fluctuating temperature treatment, perhaps it would have reduced the oviposition output of *C. rufifacies* as observed in the codling moth. Furthermore, an accelerated oviposition time (Figure 4) potentially allowed for more oviposition events (Figure 5), which resulted in the greater total egg mass observed (Figure 6). Theoretically, more eggs produced should increase the likelihood of offspring surviving in a low-quality thermal environment, assuming non-lethal temperatures, should conditions worsen after eggs are laid. For instance, Allee effects are demonstrated in blow flies, so higher larval densities may increase offspring survival [58]. Similarly, increased frequency and volume of egg production may be a different (i.e., safeguarding) bet-hedging strategy in poor thermal-quality environments [59,60]. Typically, female blow flies oviposit in large multi-female clusters [31] where the eggs in the center of this cluster receive some environmental buffering (e.g., reduced desiccation) that increases survival [61,62,63]. Our study did not track individual flies and thus cannot be certain if a few females oviposited more frequently, more total females oviposited, or both. Regardless of individual female oviposition output, *C. rufifacies* females clearly responded to acute heat stress (44 °C) by accelerating and amplifying their reproductive output at the population level.

Male blow flies exhibited similar results across all three heat shock treatments (Figure 4, Figure 5 and Figure 6). Because we did not directly investigate the effects of temperature on sperm, there are at least three possible explanations for the observed results; (1) none of the heat treatments permanently sterilized males or affected sperm production as heat-treated males successfully fertilized non-heat-treated virgin females, (2) exposure to 42 or 44 °C did temporarily sterilize or damage sperm [8], but males were able to rapidly replace sperm as seen in other blow fly species [64], or (3) heat treatments did negatively affect males (e.g., sperm death), but a few males managed to successfully mate multiple females, thus masking the effects of the heat treatments. Regardless of the mechanistic explanation, the temperature exposures were not severe enough to inhibit fertilization at the population level. Note that flies from all heat treatments produced viable eggs, but the developing larvae were discarded upon hatching, so the developmental and longevity effects of the pre-mating heat stress are unknown.

Understanding how the projected temperature shifts from climate change will impact organismal reproduction is vital for identifying vulnerable species [54,55], potential invasive species [65,66], and changes in ecosystem services [67,68]. Because some species cope with or adapt to changing temperatures better than others, there will be “winners” (i.e., survivors) and “losers” (i.e., decedents) in a warming world [69,70]. Theory suggests that tropical ectotherms, such as *C. rufifacies*, are vulnerable to rising temperatures since most are thermal specialists that already live at or near their thermal optima [71,72] and even small increases in environmental temperature may permanently push them beyond their thermal optima [9]. Thus, introduced populations of *C. rufifacies* inhabiting more temperate climates, such as North America, may initially benefit from the rising temperatures of climate change as their response to acute elevated temperatures appears to be an increase in reproductive output (i.e., more eggs). Consequently, this could lead to an ecological advantage and further displacement of native Diptera in North America [36,65,73]. Similarly, changes in oviposition timing may impact carrion ecology and succession patterns as there are demonstrated priority effects associated with *C. rufifacies* colonization of carrion [74]. Furthermore, an increased reproductive output and alteration in oviposition timing come with economic and forensic concerns. Increased oviposition by *C. rufifacies* may potentially increase cases of livestock myiasis, resulting in monetary losses from loss of product (i.e., dead or damaged livestock), increased veterinary care, and blow fly control management plans [75,76,77]. Temperature-induced changes to oviposition timing also impact the application of blow flies in forensic entomology, where forensic entomologists use the time of colonization (i.e., the onset of oviposition) in death investigations to help predict the pre-colonization interval as related to time of death [32,33]. However, if we can predict the thermal responses of *C. rufifacies* oviposition, we can better predict the potential ecological, economic, and forensic impacts of this introduced blow fly in response to climate change across environments.

## Data Availability

Data will be made available from Dryad upon the paper’s acceptance.

## Supplementary Materials

The following supporting information can be downloaded at: https://www.mdpi.com/article/10.3390/insects16030310/s1, Figure S1: Percent survival for female (grey vertical bars) and male (dashed vertical bars) at each temperature treatment ranging 10–45 °C. Survival was obswerved from 10.0–43.5 °C for all flies, while females had a greater survival than males at both 42.5 and 43.5 °C. Error bars represent the standard deviation of each sex at each individual temperature; Figure S2: (a) Oviposition frequency at each temperature treatment ranging 10–45 °C. Oviposition occurred from 22.5–42.5 °C, with the greatest frequency observed at 30.0 and 35.0 °C (both 6). Each temperature had 6 observations, and oviposition was observed 0-6 times. Fitted line is a third order polynomial line. (b) Percent oviposition at each temperature treatment ranging 10–45 °C. Oviposition occurred from 22.5–42.5 °C, with the greatest observed percentage at 30.0 and 35.0 °C (100%). Each temperature had 6 observations. Error bars represent the standard deviation of each individual temperature. Note, the standard deviation is 0.0 for both 30.0 and 35.0 °C as oviposition was 100% for both temperatures; Figure S3: (a) Individual egg masses at each temperature treatment ranging 10–45 °C. Oviposition occurred from 22.5–42.5 °C, with the greatest observed egg masses at 30.0 and 35.0 °C. Fitted lined is a fifth order polynomial line. (b) Individual egg masses at each temperature treatment ranging 10–45 °C. Oviposition occurred from 22.5–42.5 °C, with the greatest observed egg masses at 30.0 and 35.0 °C. Fitted lined is a fifth order polynomial line. Data have been jittered along the x-axis due to a high level of overlap; Figure S4: While eggs were laid from 22.5–42.5 °C, egg viability (i.e., eggs that hatched into first instar larvae) was only observed from 22.5–37.5 °C. Note, egg viability was deemed positive if any eggs laid during a temperature treatment hatched to first instars, it was not observed how many eggs from any temperature treatment hatched or if the larvae survived beyond initial hatching; File S1: Raw Data.
