# Supplementary material for: Temperature Effects on the Survival and Oviposition of an Invasive Blow Fly Chrysomya rufifacies Macquart (Diptera: Calliphoridae)
Source: Insects. 2025 Mar 17;16(3):310. doi: 10.3390/insects16030310 (PMC11943446; doi:10.3390/insects16030310)
Supplement: Supplementary file 1 [file insects-16-00310-s001.zip › Supplemental Materials Figures.pdf]

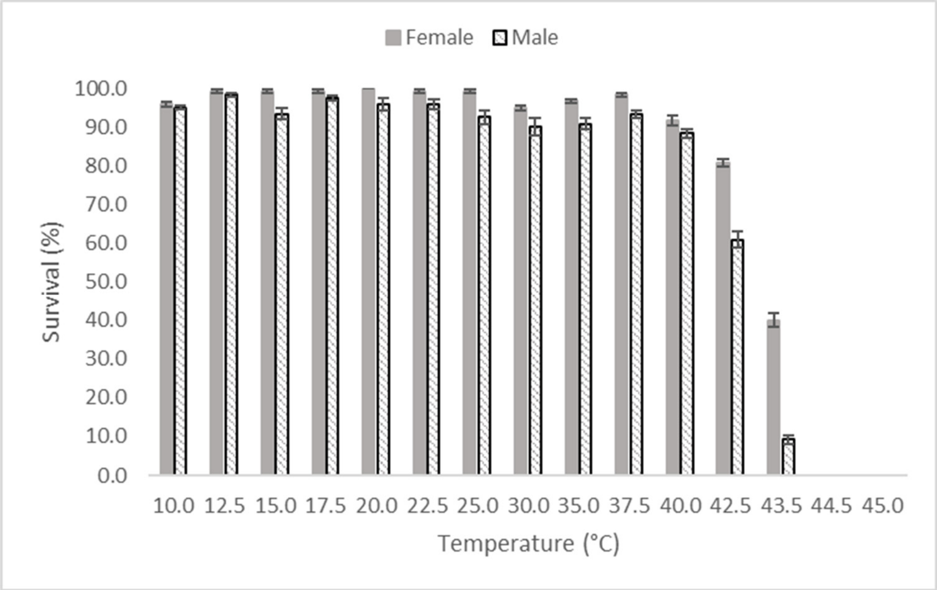

Supplemental Figure S1. Percent survival for female (grey vertical bars) and male (dashed vertical bars) at each temperature treatment ranging 10-45 °C. Survival was obswerved from 10.0 – 43.5 ˚C for all flies, while females had a greater survival than males at both 42.5 and 43.5 °C. Error bars represent the standard deviation of each sex at each individual temperature.

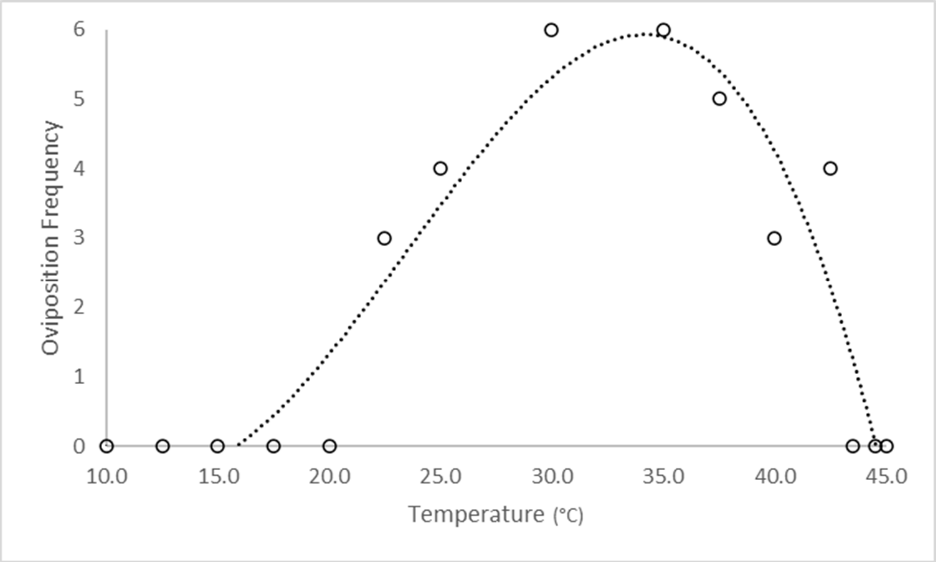

Figure S2a. Oviposition frequency at each temperature treatment ranging 10-45 °C. Oviposition occurred from 22.5 – 42.5 °C, with the greatest frequency observed at 30.0 and 35.0 ˚C (both 6). Each temperature had 6 observations, and oviposition was observed 0-6 times. Fitted line is a third order polynomial line.

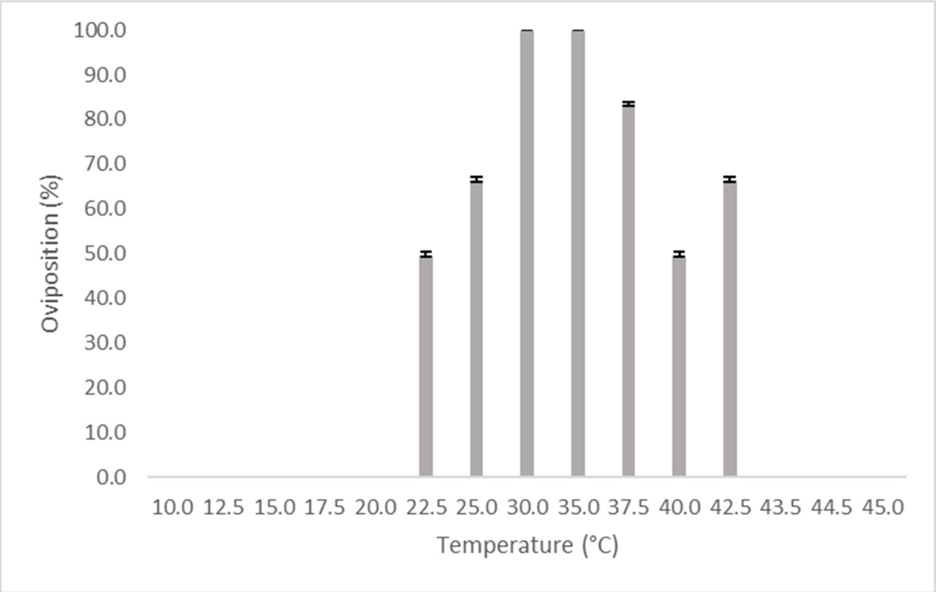

Supplemental Figure S2b. Percent oviposition at each temperature treatment ranging 10-45 °C. Oviposition occurred from 22.5 – 42.5˚C, with the greatest observed percentage at 30.0 and 35.0˚C (100%). Each temperature had 6 observations. Error bars represent the standard deviation of each individual temperature. Note, the standard deviation is 0.0 for both 30.0 and 35.0 °C as oviposition was 100% for both temperatures.

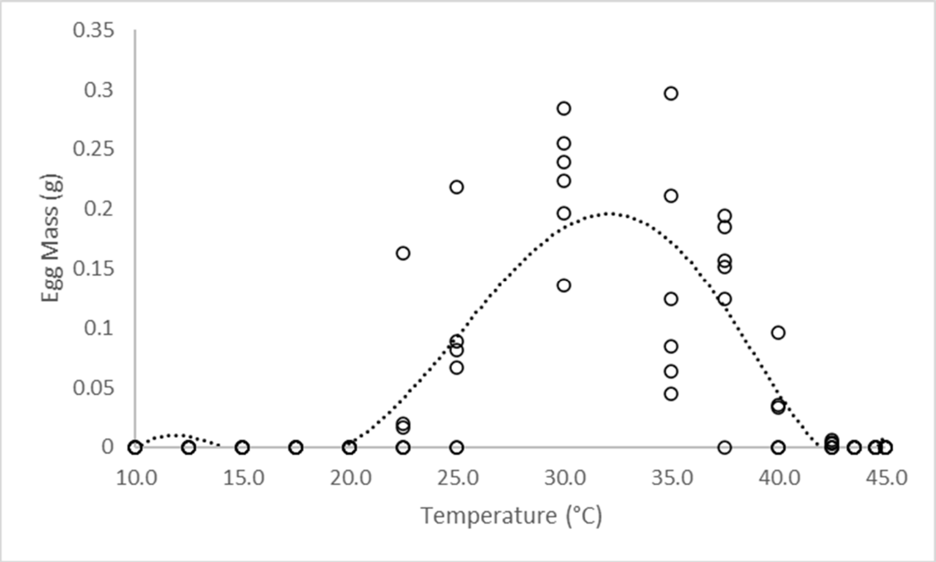

Supplemental Figure s3a. Individual egg masses at each temperature treatment ranging 10-45 °C. Oviposition occurred from 22.5 – 42.5 ˚C, with the greatest observed egg masses at 30.0 and 35.0 ˚C. Fitted lined is a fifth order polynomial line.

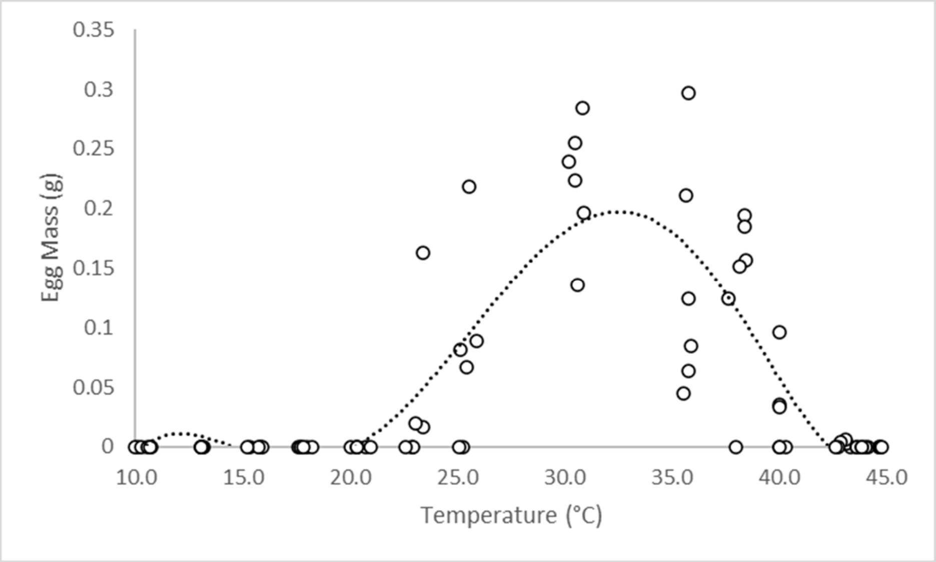

Supplemental Figure s3b. Individual egg masses at each temperature treatment ranging 10-45 °C. Oviposition occurred from 22.5 – 42.5 ˚C, with the greatest observed egg masses at 30.0 and 35.0 ˚C. Fitted lined is a fifth order polynomial line. Data have been jittered along the x-axis due to a high level of overlap.

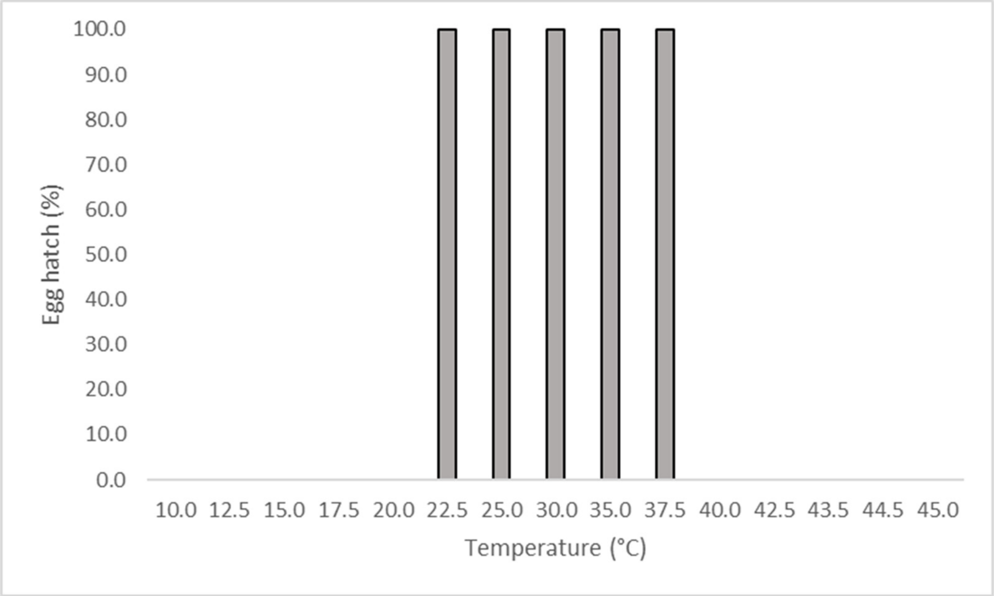

Supplemental Figure 4. While eggs were laid from 22.5 – 42.5 °C, egg viability (i.e., eggs that hatched into first instar larvae) was only observed from 22.5 – 37.5 °C. Note, egg viability was deemed positive if any eggs laid during a temperature treatment hatched to first instars, it was not observed how many eggs from any temperature treatment hatched or if the larvae survived beyond initial hatching.
